# Heave compensation prediction based on echo state network with correntropy induced loss function

**DOI:** 10.1371/journal.pone.0217361

**Published:** 2019-06-13

**Authors:** Xiaogang Huang, Dongge Lei, Lulu Cai, Tianhao Tang, Zhibin Wang

**Affiliations:** 1 Department of Electrical Automation, College of Logistics Engineering, Shanghai Maritime University, Shanghai, China; 2 College of Electrical and Information Engineering, Quzhou University, Quzhou, China; 3 Institute of Electrical Engineering, Yanshan University, Qinhuangdao, China; Beijing University of Posts and Telecommunications, CHINA

## Abstract

In this paper, a new prediction approach is proposed for ocean vessel heave compensation based on echo state network (ESN). To improve the prediction accuracy and enhance the robustness against noise and outliers, a generalized similarity measure called correntropy is introduced into ESN training, which is referred as corr-ESN. An iterative method based on half-quadratic minimization is derived to train corr-ESN. The proposed corr-ESN is used for the heave motion prediction. The experimental results verify its effectiveness.

## Introduction

When operating on sea, a vessel is inevitably affected by waves, wind and ocean currents, thereby moving away from the desired position horizontally and vertically [1]. The vertical motion of the vessel, also called heave motion, which is undesirable for offshore installations, offshore drilling and other tasks on sea because it reduces the work efficiency, causes damage to safety manufacturing system, facility and operation. To reduce this passive effects, heave compensation technologies were proposed to remove vessel’s heave motion from the load, which results in the decoupling of load motion from ship motion [2]. Now, heave compensation is popular in underwater conveying systems for oil and gas fields, payload transfer between vessels. Heave compensation technology can be classified into two classes, namely passive heave compensation (PHC) and active heave compensation (AHC). Compared to PHC, AHC can provide higher decoupling efficiency. AHC system is a close-loop system, in which the ship’s heave motion is measured and fed back to a controller. Then, the controller drives an actuator to move in an opposite direction of the heave motion. Some research result show that a controller with heave motion prediction is helpful in creating an AHC system, which results in 100% effectiveness in heave motion decoupling [2]. Furthermore, heave motion prediction can be used to partially correct a large phase lag within the controller structure [3]. Hence, heave motion prediction is an important issue to AHC.

Though it is important, the research works on heave motion prediction are not so much. In the past years, many works focused on the ship’s roll motion prediction [4, 5]. In the literature, researchers utilize autoregressive (AR), autoregressive moving average (ARMA) and moving average (MA) models to construct prediction model from time series for heave motion prediction. In [6], a heave motion model was constructed from time series based on autoregressive (AR) model, the model’s parameters were estimated using a robust estimation, i.e., iteratively reweighted least squares techniques. In [7], a method was proposed to predict vessel’s vertical motion for the purpose of forming an active compensation system. The proposed method firstly formulated a linear model of the wave-induced motion based on the dominant modes, which was obtained from fast Fourier transformation and peak detection algorithm. Then, the amplitude and phase of each mode was estimated using Kalman filter. In [1], support vector regression (SVR) combined particle swarm optimization (PSO) was adopted for heave motion prediction. In the proposed method, PSO was used to optimize the super parameter of SVR. However, the above models are only effective for linear system prediction. Since ship’s heave motion is a complex nonlinear system, it is necessary to develop a nonlinear prediction model for heave motion.

In this paper, a nonlinear model is developed for heave motion prediction based on each state network (ESN). ESN is a class of recurrent neural network (RNN), whose hidden layer (also called “dynamic reservoir”) contains many randomly and sparsely connected neural units. In ESN, only the output weights need to be trained and other weights are randomly set, and thus the training complexity of ESN is reduced. Furthermore, ESN can be found many applications in system identification and control [8, 9], wind speed and direction forecasting [10], emotion recognition [11], etc. Apart from successful applications, many research works had been done to improve the performance of ESN, which mainly focus on constructing more efficient dynamic reservoir. The leaky integrator echo state network [12], reservoirs with biological properties [13], hierarchical reservoirs [14], and simple cycle reservoir [14] are just a few examples. When training ESN, not only the basic ESN but also the improved variants, the mean square error (MSE) criteria is used. The advantage of adopting MSE is that it can obtain the least square solution and lead to training simplicity. However, MSE is sensitive to noises and outliers, and inefficient for non-Gaussian error distribution. An alternative solution to this problem is to utilize the criterion based on correntropy. Correntropy, proposed by Santamaria [15], is recognized as more flexible and robust to noise or outlier than MSE. Owing to this, a correntropy based ESN is proposed to predict the heave motion.

The rest of this paper is organized as follows. In Section 2, the basic of ESN is introduced. The correntropy based ESN is given in Section 3. The experiments for heave motion using correntropy based ESN are presented in Section 4. Finally, the concluding remarks are presented in Section 5.

## The basic of ESN

**Fig 1:**
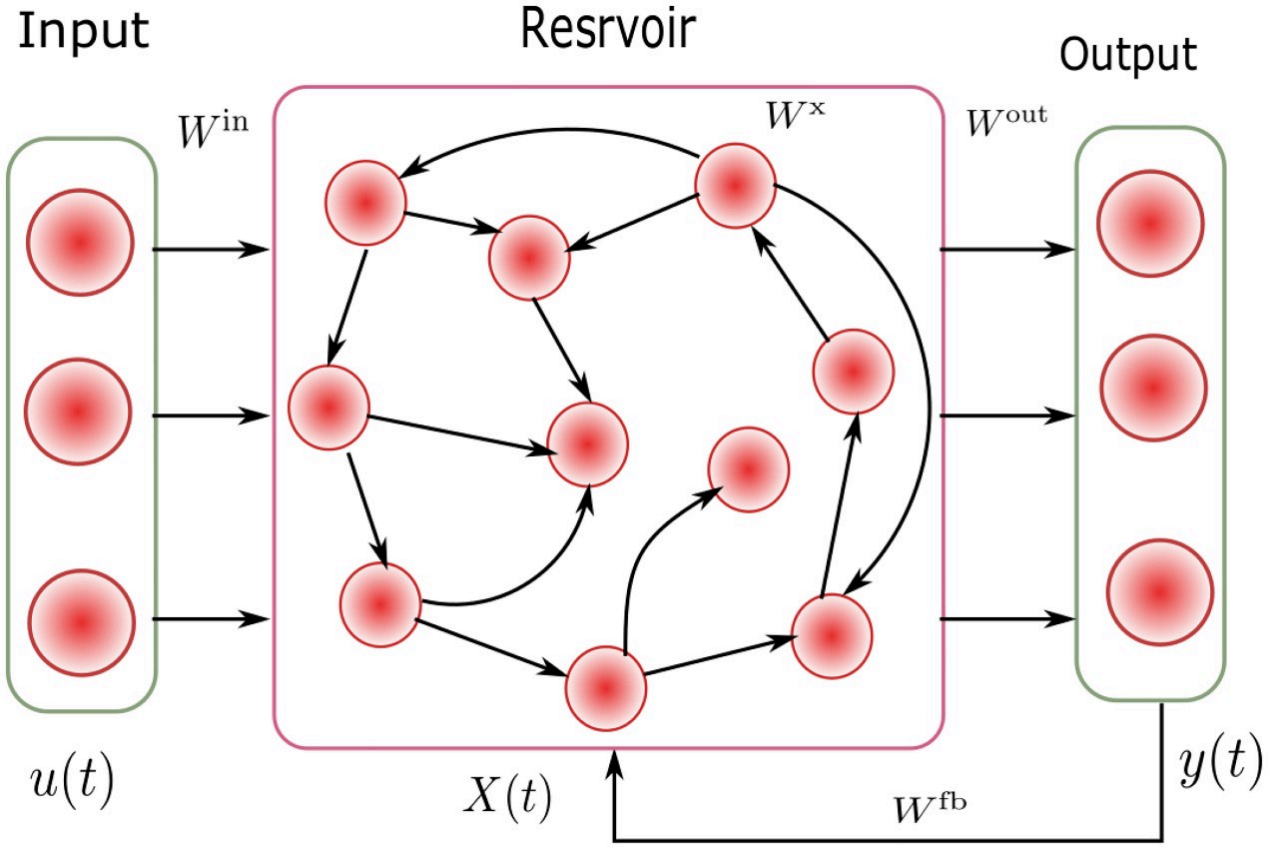
Structure of ESN.

ESN is a recurrent neural network, whose structure is shown in Fig 1. An ESN consists of an input layer, a hidden layer and an output layer. The hidden layer is also called dynamic reservoir. The neural units in reservoir are sparsely connected each other. Different from other RNNs, the input weights *W*^in^, the weights between reservoir units *W*^x^ and the feedback weight *W*^fb^ are predetermined randomly without being trained, only the output weights *W*^out^ should be trained. This characteristic greatly reduces the computation complexity. The training of ESN is divided into two stages. Firstly, the training data is fed into ESN and the state of reservoir *X*(*t*) is calculated and updated as

$$\begin{array}{c} x\left(t+1\right)=f(W^{\text{x}}X\left(t\right)+W^{\text{in}}u\left(t+1\right)), \end{array}$$ (1)

where *X*(*t*) ∈ *R*^*N*^ is the state of reservoir at time instant *t*, *u*(*t*) ∈ *R*^*L*^ is the external input at time instant *t*, *W*^x^ ∈ *R*^*N*×*N*^ is the reservoir weight matrix and *W*^in^ ∈ *R*^*N*×*L*^ is the input weight matrix. *f*(⋅) is the activation function, usually the **tanh** function is adopted. For leaky integrator ESN, the state is updated as

$$\begin{array}{c} X\left(t+1\right)=\alpha X\left(t\right)+\left(1-\alpha \right)*f(W^{\text{x}}X\left(t\right)+W^{\text{in}}u\left(t+1\right)), \end{array}$$ (2)

where *α* is called leaking rate. The output of ESN is computed as

$$\begin{array}{c} y\left(t\right)=g(z\left(t\right)W^{\text{out}}), \end{array}$$ (3)

where *y*(*t*) ∈ *R*^1×*M*^ is the output of ESN at time instant *t*, *z*(*t*) = [*X*^T^(*t*) *u*^T^(*t* + 1)] ∈ *R*^1×(*N*+*L*)^ is the concatenation of reservoir states and input vectors and *W*^out^ ∈ *R*^(*N*+*L*)×*M*^ the output weight matrix, *g*(⋅) is a nonlinear mapping function. In practice, the nonlinear function *g*(⋅) is selected as linear function. Therefore, the output can be written as

$$\begin{array}{c} y\left(t\right)=z\left(t\right)W^{\text{out}}. \end{array}$$ (4)

Let

$$\begin{array}{c} Y=[\begin{array}{c} y(t)\\ y(t+1)\\ \vdots \\ y(t+N-1) \end{array}],Z=[\begin{array}{c} z(t)\\ z(t+1)\\ \vdots \\ z(t+N-1) \end{array}], \end{array}$$ (5)

one can get

$$\begin{array}{c} Y=ZW^{\text{out}}, \end{array}$$ (6)

then, the optimal output weight matrix is obtained as

$$\begin{array}{c} W^{\text{out}}={\left(Z^{T}Z\right)}^{-1}Z^{T}Y. \end{array}$$ (7)

## Correntropy based ESN

### Correntropy

In information theoretic learning (ITL), correntropy is a local similarity measure between two random vectors. It is regarded as a generalization of correlation function. Given two arbitrary random variables A and B, their correntropy is defined as

$$\begin{array}{c} V_{\sigma }\left(A,B\right)=E\left[k_{\sigma }\left(A,B\right)\right], \end{array}$$ (8)

where *k*_*σ*_(⋅) is the kernel function that satisfies Mercer’s theorem with kernel size *σ* and *E*[⋅] denotes the mathematical expectation. In practice, only a finite number of samples are available and the real joint probability density of *A* and *B* is unknown. Therefore, the mathematical expectation is approximated by arithmetical average as

$$\begin{array}{c} {\hat{V}}_{\sigma }\left(A,B\right)=\frac{1}{M}\sum_{i=1}^{M}k_{\sigma }\left(a_{i},b_{i}\right). \end{array}$$ (9)

In this paper, the Gaussian kernel function is selected, its expression is as follows

$$\begin{array}{c} k_{\sigma }\left(a_{i},b_{i}\right)≜G\left(a_{i},b_{i}\right)=\text{exp}(-\frac{{\left(a_{i}-b_{i}\right)}^{2}}{2\sigma^{2}}). \end{array}$$ (10)

Therefore, Eq (9) can be rewritten as

$$\begin{array}{c} {\hat{V}}_{\sigma }\left(A,B\right)=\frac{1}{M}\sum_{i=1}^{M}\text{exp}(-\frac{{\left(a_{i}-b_{i}\right)}^{2}}{2\sigma^{2}}). \end{array}$$ (11)

The closer between *A* and *B* is, the larger the correntropy is. Compared with MSE, correntropy is not sensitive to noises or outliers and lead to more robust estimation.

### Training ESN based on regularized correntropy criterion

In the training phase of ESN, the MSE is replaced by correntropy in order to improve the performance of ESN. Furthermore, to enhance the generalization of ESN, a *L*_2_ norm regularization term is added. The new criteria for training ESN is given as

$$\begin{array}{c} J\left(W^{\text{out}}\right)=\max_{W^{\text{out}}}[\sum_{i=1}^{M}\text{exp}(-\frac{\|y\left(i\right)-z\left(i\right)W^{\text{out}}{\|}^{2}}{2\sigma^{2}})-\gamma {\left\|W^{\text{out}}\right\|}^{2}]. \end{array}$$ (12)

The new criteria (12) is not quadratic any more. In this paper, the half-quadratic optimization is used to solve the optimization problem (12).

**Proposition 1** *For* $G\left(z\right)=\text{exp}(-\frac{{\left\|z\right\|}^{2}}{2\sigma^{2}})$, *there exists a convex conjugated function φ, such that*

$$\begin{array}{c} G\left(z\right)=\underset{\alpha \in R^{-}}{\sup}(\alpha \frac{{\left\|z\right\|}^{2}}{2\sigma^{2}}-\varphi \left(\alpha \right)). \end{array}$$ (13)

*Moreover, for a fixed **z**, the supremum is reached at α* = −*G*(***z***).

Hence, introducing (13) into the objective function (12), the following augmented objective function can be obtained,

$$\begin{array}{c} \hat{J}\left(W^{\text{out}},\alpha \right)=\max_{W^{\text{out}},\alpha }[\sum_{i=1}^{M}(\alpha_{i}\frac{\|y\left(i\right)-z\left(i\right)W^{\text{out}}{\|}_{2}^{2}}{2\sigma^{2}}-\varphi \left(\alpha_{p}\right))-\lambda {\left\|W^{\text{out}}\right\|}^{2}], \end{array}$$ (14)

where ***α*** = (*α*_1_, *α*_2_, …, *α*_*M*_) stores the auxiliary variables appeared in the half-quadratic optimization. Moreover, for a fixed *W*^out^, the following equation holds

$$\begin{array}{c} J\left(W^{\text{out}}\right)=\hat{J}\left(W^{\text{out}},\alpha \right). \end{array}$$ (15)

The optimal problem (14) can be solved via the following iterative manner,

$$\begin{array}{c} \alpha_{i}^{\tau +1}=-\text{exp}\{-\frac{\|y\left(i\right)-z\left(i\right)W^{\text{out}}{\|}^{2}}{2\sigma^{2}}\}, \end{array}$$ (16)

and

$$\begin{array}{c} W_{\tau +1}^{\text{out}}=\arg\max_{W^{\text{out}}}{\left(ZW^{\text{out}}-Y\right)}^{T}\Lambda \left(ZW^{\text{out}}-Y\right)-\gamma W^{\text{out}}W^{\text{out}T} \end{array}$$ (17)

where *τ* denote the *τ*th iteration and Λ is a diagonal matrix with its primary elements $\Lambda_{ii}=-\alpha_{i}^{\tau +1}$. The optimal problem (17) is easy to be solved, one can set the partial derivative of $\hat{J}\left(W^{\text{out}},\alpha \right)$ with respective to *W* to zero, and yields

$$\begin{array}{c} \frac{\partial \hat{J}\left(W^{\text{out}},\alpha^{\tau +1}\right)}{\partial W^{\text{out}}}=2\left(Z^{T}\Lambda Z-\gamma I\right)W^{\text{out}}-2Z^{T}\Lambda Y=0. \end{array}$$ (18)

Therefore,

$$\begin{array}{c} W_{\tau +1}^{\text{out}}={\left(Z^{T}\Lambda Z-\gamma I\right)}^{-1}Z^{T}\Lambda Y. \end{array}$$ (19)

After some iterations, the objective function (14) converges. The strict proof can be referred to [16].

## Experimental results

**Fig 2:**
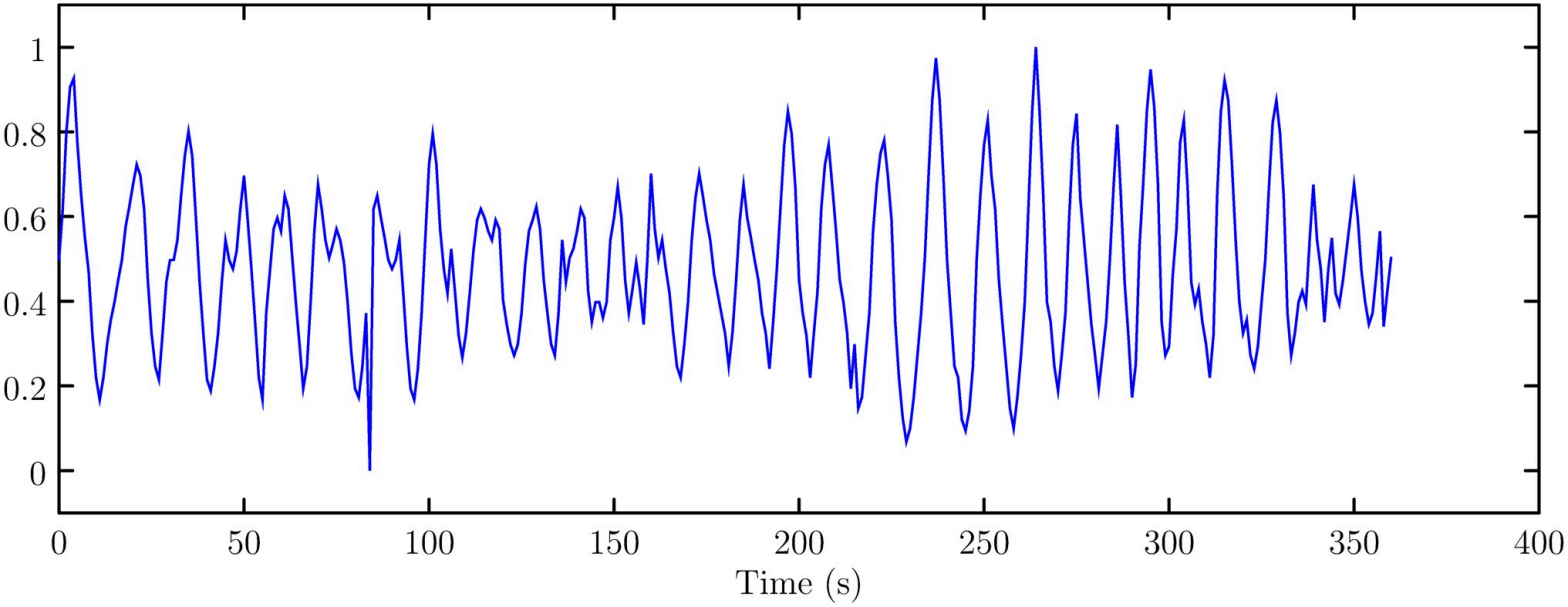
Heave motion data.

In this section, the simulation studies are conducted to verify the effectiveness of the proposed method. All the algorithms are implemented in Matlab 2016b programming language and run in a ThinkPad T440 notebook computer with Intel Core™ i5-4200U processor, 8G random access memory (RAM). The heave motion data is taken from [17]. The data is measured from a simulation platform of wave movement with an accelerometer. The sampling frequency is 100Hz. The measured data is normalized into [0, 1], which is shown in Fig 2 (The data can be referred to S1 Fig).

### Data preprocessing

The measured data contains many components including sudden vibration, high frequency component generated by fluctuating oil pressure and direct component [18]. Therefore, the original data is preprocessed by a simple filtering operation. The frequency domain filtering method based on Fast Fourier Transform (FFT) is applied to the original measured signal. The filtering is performed in frequency domain. A low-pass filter between 0 and 15 Hz is adopted and its frequency response is as follows,

$$\begin{array}{c} H\left(f\right)=\{\begin{array}{cc} 1, & 0<f<15\\ 0, & \text{others} \end{array} \end{array}$$ (20)

**Fig 3:**
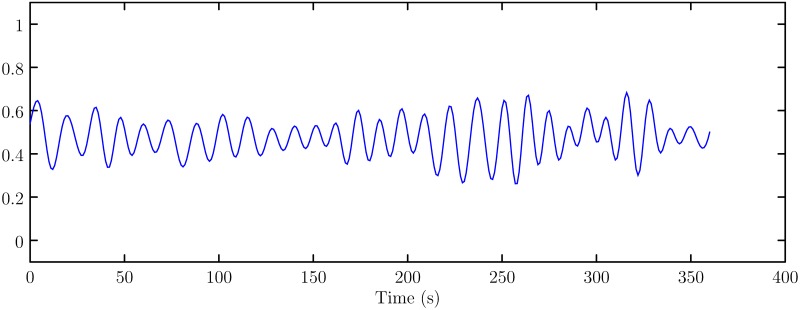
Heave motion data after filtering.

The filtered data is shown in Fig 3 (The data can be referred to S2 Fig).

### Prediction results

The filtered data is used for prediction experiments. Let *y*(*k*), *k* = 1, 2, ⋯, *n* denotes the heave motion time series. In this paper, a three-order model is used for prediction, i.e., *y*(*k* − 1), *y*(*k* − 2), *y*(*k* − 3) is used to predict *y*(*k*). For the purpose of comparison, an autoregression (AR) model and original ESN are also implemented and used for prediction. The RMSE (root mean square error) is used to evaluate the prediction performance, which is defined as

$$\begin{array}{c} \text{RMSE}=\sqrt{\frac{\sum_{k=1}^{N}{\left[y\left(k\right)-\hat{y}\left(k\right)\right]}^{2}}{N}}, \end{array}$$ (21)

where *y*(*k*) is the real measured value at time *k* and $\hat{y}\left(k\right)$ is the predicted value, *N* is the number of data points used for training or test.

**Fig 4:**
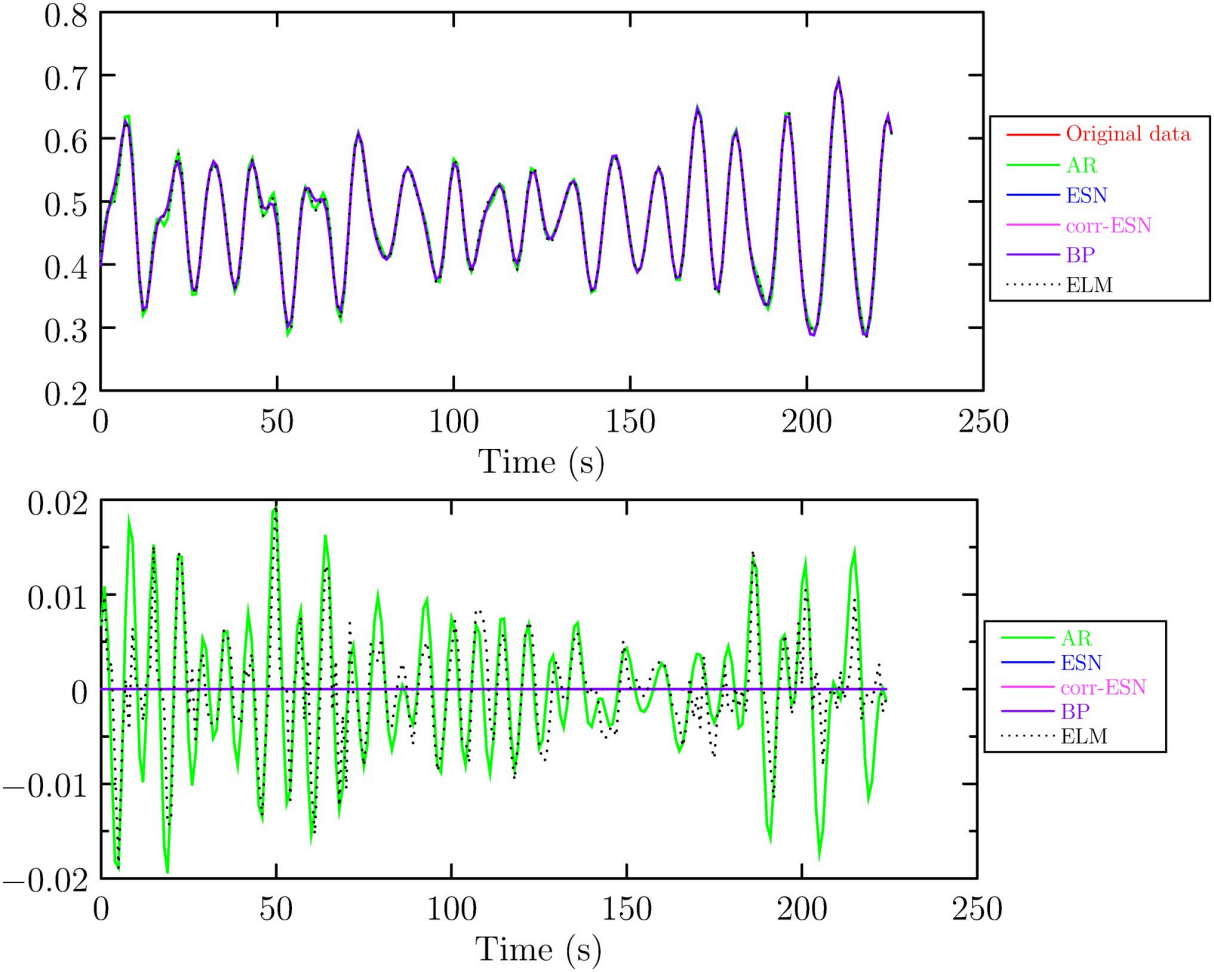
The training results and error. Top: training result, Bottom: the training error.

**Fig 5:**
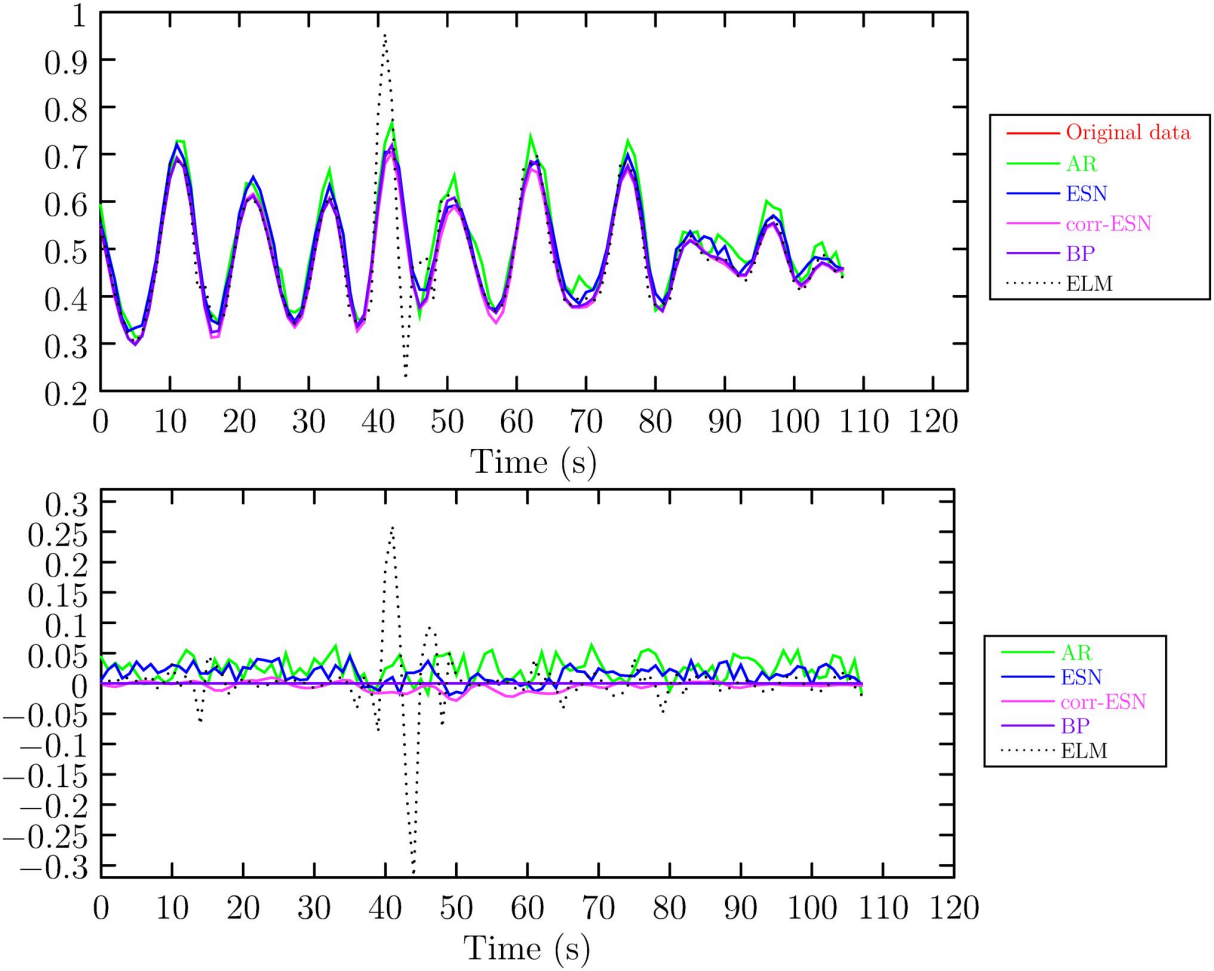
The one-step prediction results and error. Top: prediction result, Bottom: the prediction error.

In experiments, the whole data is partitioned into two parts, the first 70% is used for training and the rest 30% is used for testing. The parameters of ESN are set as follows. The size of reservoir is 1000, λ in (17) is set to 10^−6^ and *σ* in (10) to 0.1. The maximum number of iteration in correntropy based ESN is 200. If the error of objective function value between successives iteration is less than a given tolerance, then the iterations break out and the algorithm terminates. The tolerance is set to 10^−5^. The training results and error are shown in Fig 4 (The data can be referred to S3 Fig). Fig 5 (The data can be referred to S4 Fig) shows the one-step prediction results and prediction error. It can be seen that the training and prediction error of the proposed method are the smallest.

To objectively evaluate the performance of the referenced methods, Table 1 lists the RMSE in training and prediction phase of the referenced methods.

**Table 1:** RMSEs in training and prediction phase of different methods.

	AR	ESN	corr-ESN	BP	ELM
Trainging	0.0073	5.7923E-6	3.8741E-13	0.0067	0.0056
Predictioin	0.0310	0.0206	0.0088	0.0224	0.0531

## Conclusions

A correntropy based ESN is proposed to predict heave motion for the purpose of heave compensation. The proposed approach adopts correntropy instead of MSE as the error criterion for ESN training, which is called corr-ESN. An iterative training algorithm is derived using half quadratic optimization theory. Since the correntropy is insensitive to noise and outliers, the corr-ESN is more accurate than ESN for heave motion prediction. Simulation results validate the effectiveness of the proposed method.

## Supporting information

S1 Fig The data used to plot the Fig 2. (CSV)

S2 Fig The data used to plot the Fig 3. (CSV)

S3 Fig The data used to plot the Fig 4. (CSV)

S4 Fig The data used to plot the Fig 5. (CSV)
